# The Royal Veterinary School at Budapest, Hungary, as at Present Organized*Oesterreichische Vierteljahresschift für Veterinairkunde, vol. 60.

**Published:** 1884-04

**Authors:** 


### THE ROYAL VETERINARY SCHOOL AT BUDAPEST, HUNGARY,

### AS AT PRESENT ORGANIZED.*

WE MAKE no apology to our readers for offering to their consideration a translation upon the above subject, for every contribution of the kind possesses both historical and practical importance.

The Veterinary School at Budapest bears the above mentioned title, and ranks as an academy among the educational institutions of the land. It is directly under the supervision of the Ministers of Agriculture, Industry and Commerce.

The purpose of the institute is to educate Veterinarians in all those branches of education which are requisite to a thorough understanding of their professional duties, and to give instruction to those who wish it in single branches of Veterinary education. Further, it is a part of the work of said institute to advance the animal hygienic system of the land in every possible way by means of carefully conducted experiments and exact observations, as well as to advance forensic medicine and everything pertaining to Veterinary science.

#### THE STUDENTS.

The following classes of students are accepted :

* Oesterreichische Vierteljahresschift für Veterinairkunde, vol. 60.

1st. Those who desire to take the complete course in order to become Veterinarians.

2d. Hospitants or hearers of special lectures, but who do not intend to become practitioners.

3d. Horseshoers; that is, persons that intend to become such, but not to become Veterinarians.

EXPENSE.

The students receive free instruction, with the exception of a fee of four florins to the recorder.

LANGUAGE.

The lectures are delivered in the Hungarian language, but the students not sufficiently familiar with it may be examined in any other language which the teachers can understand.

COURSE FOR VETERINARIANS.

Any person having the necessary educational and moral qualifications, that is not under 17 years old, will be accepted as a student, after having passed a matriculatory examination. Graduates from certain classes in the public schools are accepted without the above examination.

This examination consists in a correct knowledge of the Hungarian language; in a knowledge of the geography and history of the world, and especially that of Hungary and Austria; and in a knowledge of the general principles of mathematics. Notice to take part in the matriculation must be given the direction on the 25th of August, and the candidate appear in person on September 1st.

The educational course extends over three years or six sessions. The school begins on September 1st, and ends on June 15th of the succeeding year.

SUBJECTS TAUGHT.

*Natural scientific or preparatory course.*

Mineralogy, Botany, Zoology, Physics and Chemistry.

*Technical Branches.*

Anatomy—Descriptive and Topographical; Physiology and Histology (with laboratory work); Pathological Anatony (with laboratory work); General Pathology, Therapeutics (in all its forms); Breeding (which includes lectures in soundness, dietetics, race and buying, selling and transport); Theoretical and

Practical Horseshoeing; special Pathology and Therapeutics, Internal Clinical practice,Surgical Pathology and Therapeutics, Obstetrics, Surgical Clinic, lectures upon instruments and bandaging, Dog Clinic, Forensic Veterinary Medicine, Veterinary Police and Contagious Diseases, Meat Inspection, History of Veterinary Medicine, visits to animals outside the school for special instruction.

The instruction is arranged as follows :

FIRST YEAR.

*Winter (1st) Session.*

Mineralogy, Zoology, Chemistry, Theoretic Horseshoeing, each three hours a week ; Physic, two hours a week ; Descriptive and Topographical Anatomy, Anatomical Practice, and Practical Horseshoeing, each five hours a week.

*Summer (2d) Session.*

Botany, two hours a week ; Descriptive and Topographical Anatomy, Physiology and Histology, and Practical Horseshoeing, each five hours a week; Practice in Laboratory in Histology, six hours a week; Exterior and Breeding of Horses, and Examination for Soundness, each three hours a week.

SECOND YEAR.

*Winter (3d) Session.*

Physiology and Histology, Theoretic Pathological Anatomy, and Breeding Sheep and Cattle, each three hours a week; General Pathology and Dietetics, each three hours a week; Practice in Laboratory, Exercises in Autopsies, Visiting Clinic for Internal Diseases, Visiting Clinic for Surgical Diseases, and Practice in Dissection, each five hours a week.

*Summer (4th) Session.*

Exercises in Autopsical Laboratory, Practice in Histology, Therapeutics, Pharmaceutical Exercises, Visits to Internal Clinic and Visits to Surgical Clinic, each five hours a week; Obstetrics and Instrumental and Bandage Practice, each two hours a week.

THIRD YEAR.

*Winter (5th) Session.*

Special Pathology and Therapeutics, Internal Clinic, Theoretic Aciurgery and Practice, Surgical Clinic, Practical

Pathology in Laboratory, Veterinary Police, etc., and Dog Clinic, each five hours a week; Horse-breeding and Stud Management, each three hours a week; Free Clinic.

*Summer (6th) Session.*

Special Pathology and Therapeutics, Internal Clinic, Theoretic Surgery and Operative Practice, Special Surgical Pathology, Autopsy Exercises, Veterinary Police, etc., Forensic Medicine, and Dog Clinic, each five hours a week; Animal Transport, Market Inspection, etc., each two hours a week; Free Clinic.

During the third year the students also visit sick animals outside the School, especially when enzootic diseases prevail. Each student has personal charge of patients in the hospital.

Any student that has heard the lectures upon the natural-scientific branches at a recognized school, may be excused from attending them at the Veterinary School.

Foreigners can receive the Diploma of the School, but cannot practice in Hungary, except with the permission of the Government.

Special courses are arranged for what are called Veterinary Farriers and Horseshoers.

The faculty consists of nine professors and seven assistants. The number of students present during the year 1881–2 was 173.

